# Negative effects of abamectin on soil microbial communities in the short term

**DOI:** 10.3389/fmicb.2022.1053153

**Published:** 2022-12-05

**Authors:** Danyan Qiu, Nuohan Xu, Qi Zhang, Wenya Zhou, Yan Wang, Zhenyan Zhang, Yitian Yu, Tao Lu, Liwei Sun, Ning-Yi Zhou, W. J. G. M. Peijnenburg, Haifeng Qian

**Affiliations:** ^1^College of Environment, Zhejiang University of Technology, Hangzhou, China; ^2^College of Environment and Ecology, Xiamen University, Xiamen, China; ^3^State Key Laboratory of Microbial Metabolism, School of Life Science and Biotechnology, Shanghai Jiao Tong University, Shanghai, China; ^4^Institute of Environmental Sciences (CML), Leiden University, Leiden, Netherlands; ^5^National Institute of Public Health and the Environment (RIVM), Center for Safety of Substances and Products, Bilthoven, Netherlands

**Keywords:** abamectin, microorganism, ecotoxicity, antibiotic resistance genes, soil microbial community

## Abstract

With the widespread use of abamectin in agriculture, there is increasing urgency to assess the effects of abamectin on soil microorganisms. Here, we treated plant–soil microcosms with abamectin at concentrations of 0.1 and 1.0 mg/kg and quantified the impacts of abamectin on bulk and rhizosphere soil microbial communities by shotgun metagenomics after 7 and 21 days of exposure. Although abamectin was reported to be easily degradable, it altered the composition of the soil microbial communities, disrupted microbial interactions, and decreased community complexity and stability after 7 days of exposure. After treatment with abamectin at a concentration of 1.0 mg/kg, some opportunistic human diseases, and soil-borne pathogens like *Ralstonia* were enriched in the soil. However, most ecological functions in soil, particularly the metabolic capacities of microorganisms, recovered within 21 days after abamectin treatment. The horizontal and vertical gene transfer under abamectin treatments increased the levels of antibiotic resistance genes dissemination. Overall, our findings demonstrated the negative effects of abamectin on soil ecosystems in the short-term and highlight a possible long-term risk to public and soil ecosystem health associated with antibiotic resistance genes dissemination.

## Introduction

Intensified global agriculture has increased agrochemical inputs in the past decades (Tramberend et al., 2019; Jiao et al., 2020; Yuan, S. et al., 2021). The annual usage of biopesticides increased by more than 15%, outpacing that of chemical pesticides (Marrone, 2014). As a typical biopesticide, abamectin is produced commercially by the soil bacterium *Streptomyces avermitilis*. Abamectin is biodegradable, has a short field re-entry, and low risk to beneficial insects (Marrone, 2019). Abamectin is one of the most used agents in agriculture in the prevention of soil-borne diseases (Kolar et al., 2008). Since 2007, abamectin has been widely used in the control of rice pests in China due to its strong insecticidal activity (He et al., 2007; El-Saber Batiha et al., 2020). Although abamectin degrades rapidly, has little toxicity to crops, and is not likely to remain in the soil, the toxicity of abamectin to soil microorganisms is still unknown despite its widespread use.

The soil ecosystem is globally among the most biodiverse environmental compartments and undertakes multiple service functions (Wall et al., 2015; Wittwer et al., 2021; Zhang et al., 2022; Zheng B. et al., 2022; Zheng X. et al., 2022). Soil microorganisms are the executors of the ecological service functions, which can help crop growth by improving soil structure and soil nutrient cycling (Bahram et al., 2018; Qu et al., 2020; Ray et al., 2020; Zhang et al., 2022a). Therefore, the stability of soil microbial communities is one of the critical factors in maintaining soil ecosystem function (Ramirez et al., 2018). In agricultural production, pesticides inevitably remain in the soil, affecting rhizosphere microorganisms and plant growth (Kepler et al., 2020). Pesticides can be absorbed by plant roots and transferred to other above-ground part of the plant (Xu et al., 2020; Qu et al., 2021). Rice (*Oryza stiva* L.) is an important land crop plant and feeds over 50% of the global population (Ge et al., 2012). The microorganisms colonizing the rice rhizosphere (immediately surrounding the root) contribute among others to rice growth, soil structure, and pathogen suppression (Dennis et al., 2010; Lundberg et al., 2012). Residues of abamectin and abamectin metabolites in soil may adversely affect soil invertebrates and the roots of some crops like cucumber (Kolar et al., 2008; Huang et al., 2018). Additionally, the genus *Streptomyces* that produces abamectin is also the host of antibiotic resistance genes (ARGs; Majer et al., 2021). Abamectin thus plays a role in the development and spread of antibiotic resistance in plant–soil ecosystems.

With the aim of assessing the ecotoxicity of abamectin in a plant–soil ecosystem, we selected the model crop rice to construct a plant–soil ecosystem and we simulated exposure to abamectin. We explored the impacts of abamectin on the diversity, composition, community stability, and functions of soil microbial communities, and the ARGs dismission by using shotgun metagenome analysis. Our aims with regard to the research in the plant–soil ecosystem were to: (1) identify the effects of abamectin treatment on microbial community diversity, composition, and stability; (2) determine how abamectin affects the pathogens associated with opportunistic human and soil-borne diseases; (3) determine how abamectin affects the functions of microorganism; and (4) quantify the abundance and transfer of ARGs during the study. Accurately evaluating the contribution and ecological risks of abamectin on plant–soil ecosystems can guide the wide use of eco-friendly biopesticides.

## Materials and methods

### Establishment of plant–soil-microorganism microcosms

Rice (*Oryza sativa* L. *Indica Yazhan*) seeds were soaked in 0.6% nitric acid solution for 10 min to release them from dormancy for effective germination. The seeds were then disinfected with 75% ethanol for 1 min, followed by 2.5% calcium hypochlorite for 5 min each, and rinsed 6–7 times with sterile water. The surface-sterilized seeds were placed in the dark at 30°C for 7 days for germination, after which germinated rice seedlings were transplanted into a plastic pot containing about 230 g of soil and 30 g of sterilized water. A soil suspension made from paddy soil (from 27° 17′ 27.70″ N, 119° 56′ 50.70″ E) was then added to a plastic pot as the source of rhizosphere microorganisms (soil:sterile water = 30:200). The obtained plant–soil-microorganism microcosms were placed in a greenhouse with cool-white fluorescent lights with a 300 μmol/(m^2^·s) fluorescence intensity at day, temperatures of 25 ± 0.5°C and 80% relative humidity, a 12-h light/12-h dark cycle for 4 weeks.

### Abamectin treatment of plant–soil-microorganism microcosms

Abamectin was purchased from Aladdin (Shanghai). The initial solution was diluted with deionized water into abamectin solutions with final concentrations of 0.1 and 1.0 mg/kg, respectively, and added to the plant–soil-microbiome microcosms after 14 days of rice growth. An equal amount of deionized water was added to the control group. Every 2 days 120 ml deionized water was added to the replicates of each microcosm to keep them submerged.

### The collection of soil samples

Bulk and rhizosphere soils in which rice was planted, were collected 7 and 21 days after seedling transplantation (the suspension of the paddy soil was sampled on day 0). The rice was carefully uprooted, and the bulk soil was removed by shaking the roots. The upper soil was gently collected with a spoon as bulk soil to explore the direct effects of abamectin treatment on the soil microbial community. The soil remaining on the rice roots (approximately 1 mm along the root surface) was regarded as the rhizosphere (Edwards et al., 2015). The roots with rhizosphere soil were transferred to a centrifuge tube containing sterile phosphate buffer saline (PBS; pH 7.3–7.5). The centrifuge tubes were shaken at 180 rpm for 20 min on an orbital shaker. Then, the roots were removed, and the mixed solution was centrifuged at 4,500 rpm for 20 min. The supernatant was removed, and the rhizosphere soil was collected (Bulgarelli et al., 2012) to explore the direct effects of abamectin treatment on the rhizosphere microbial community. Finally, all soil samples were stored at −80°C immediately until analysis.

### Microcosm metagenome sample preparation and sequencing

Metagenomic analysis of bulk soil microorganisms and rhizosphere soil microorganisms after abamectin treatments were performed in three replicates at 7 and 21 days. Total genomic DNA of these samples was isolated from 0.5 g of soil per sample using a DNA extraction kit (NEXTFLEX™ Rapid DNA—Seq Kit, Shanghai Majorbio Bio-pharm Technology Co., Ltd., Shanghai, China) according to the manufacturer’s instructions. The Qubit 2.0 (Thermo Fisher Scientific, Waltham, United States) and NanoDrop One (Thermo Fisher Scientific, Waltham, United States) were used to determine the concentration and purity of the extracted soil DNA, respectively. Sequenced libraries were generated using a DNA library preparation kit according to the manufacturer’s instructions (NEB Next Ultra DNA Library Preparation Kit, New England Biolabs, MA, United States), and the library quality was verified using Qubit 3.0 Fluorometer (Life Technologies, Grand Island, NY). Next, the Illumina NovaSeq platform (Illumina, CA, United States) was used to perform DNA sequencing.

### Microcosm metagenome assembly and analysis

Filtering of low-quality bases in the metagenome dataset (Trimmomatic v0.36) resulted in clean data that was used for subsequent analyses (Bolger et al., 2014). Next, the MEGAHIT v1.0.6 performed the *de novo* assembly of clean data (Li et al., 2015), and Prodigal^1^ predicted each assembled scaftig (>500dp) to open reading frames (ORFs). At the same time, redundant ORFs were removed using CD-HIT v4.7, resulting in unique initial gene (unigene) clusters (95% concordance, 90% coverage; Fu et al., 2012), and each cluster took the longest sequence as its representative. Additionally, clean data were mapped to unigenes using BBMap to obtain the number of reads to which unigenes were mapped in each sample. The abundance of unigene was calculated with RPKM (mapped reads per kilobase per million reads):


(1) $${\mathrm{RPKM}}_{i}=\frac{x_{i}}{L_{i}\;\left(\mathrm{KB}\right)\times x_{total}\;\left(\mathrm{millions}\right)}$$

Where *x_i_* was the number of mapped reads for unigene*_i_* in each sample, *x_total_* was the total number of all mapped reads in each sample, and L*_i_* was the sequence length of unigene*_i_*.

The nonredundant (NR) database of NCBI and the Kyoto Encyclopedia of Genes and Genomes (KEGG, http://www.kegg.jp/kegg/) were employed to perform a BLAST search for taxonomic and functional annotations using the DIAMOND software,^2^ respectively. Also, the ARGs were identified by DIAMOND in the Comprehensive Antibiotic Resistance Database (CARD) mentioned above at e-value < 1e^−5^. The LCA algorithm in MEGAN was utilized to calculate the abundance and each taxonomic classification (kingdom, phylum, class, order, family, genus, species) based on the alignment of NR unigenes with an e-value ≤ 1e^−10^ (Huson et al., 2007). Opportunistic human pathogens were searched from an online database.^3^

### Statistical and visualization

The alpha diversity index of Shannon and Richness were calculated using the R vegan and picante package (version 4.0.3). Principal co-ordinates analysis (PCoA) plots were generated from the Bray-Curtis distance created using the R package ggplot2 and vegan (version 4.0.3). ANOSIM analysis (transformed data by Bray-Curtis, permutation = 999) was used to determine if beta diversity differed between abamectin-treated soil and the terrestrial control groups. All bar and heatmap charts were designed by R package ggplot2 (version 4.0.3). The *p* value of the Spearman’s correlation in network analysis was amended using the Benjamini-Hochberg’s FDR (false discovery rate) method to avoid false-positive results. The Spearman correlations (positive correlation: R > 0; positive correlation: R < 0; *p* < 0.05) of the microorganisms and multiple abamectin treatments were calculated using the R psych package. The networks were drawn using the software Gephi (version 0.9.2). The remaining results are presented as the mean ± standard deviation (SD). Using the R function kruskal.test (version 4.0.3) to evaluate Significant differences (*p* < 0.05) by the Kruskal–Wallis’s test.

## Results

### Effect of abamectin on diversity, composition, and stability of the soil microbial community

**Figure 1:**
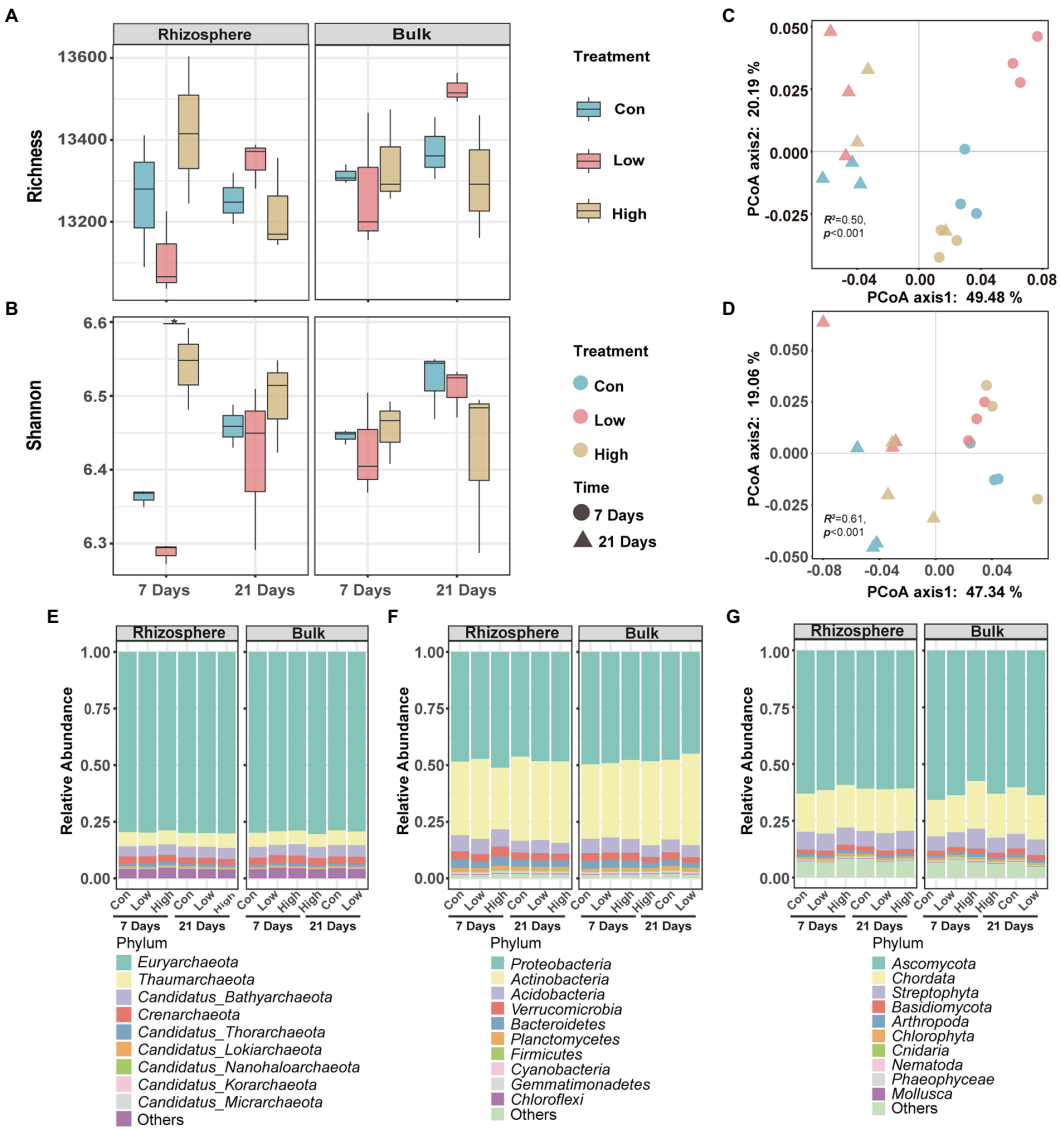
Effects of abamectin on diversity and structure of microbial communities. **(A,B)** Shannon index and richness calculated at the genus level. * Represents the significant differences between control and abamectin treatments (Kruskal–Wallis’s test, *p* < 0.05). **(C,D)** PCoA of Bray–Curtis dissimilarities of microbial communities at the genes level. Statistical significance was evaluated *via* PERMANOVA test. **(E–G)** Effects of abamectin on the community composition of the main phyla related with eukaryote, bacteria, and archaea, respectively.

The metagenome was used to characterize the soil microbial community diversity and composition after abamectin treatment. Alpha diversity analysis can reflect the richness and diversity of microbial communities. The greater the values of the Shannon and Simpson indices, the higher the degree of diversity of the microorganism community (Xu et al., 2020). The Shannon index of the rhizosphere microorganisms increased significantly with the two tested concentrations of abamectin for 7 days, but no effects were detectable on other soil microbial communities (Figures 1A,B).

PCoA with Bray–Curtis’s dissimilarity of the genera abundance indicated that both concentrations of abamectin significantly impacted the diversity of the microbial community in the soil during the 7 and 21 days of exposure (Figures 1C,D). Furthermore, the dissimilarity of microbial communities after 7 days of exposure was generally higher than the dissimilarity of microbial communities after 21 days of exposure (Supplementary Figure S1). This indicated that abamectin treatment disrupted the structure of the soil microbial community within 7 days of exposure. The soil microbial community gradually recovered the microbial succession after 21 days of exposure.

There were significant differences in the composition of the bulk and rhizosphere microbial communities after the abamectin treatment (Figures 1E–G). The analysis of the significant effects of abamectin on the relative abundance of microbial phyla showed: (1) an increase in the abundance of *Actinobacteria* in the rhizosphere microbial community after the low abamectin treatment and a decrease of *Actinobacteria* after the high abamectin treatment during the first 7 days of exposure, with opposite effects recorded after 21 days (Supplementary Figures S2A,B); (2) After abamectin treatment, the dominant phyla that changed significantly in different groups were different from each other (Supplementary Figure S2).

**Figure 2:**
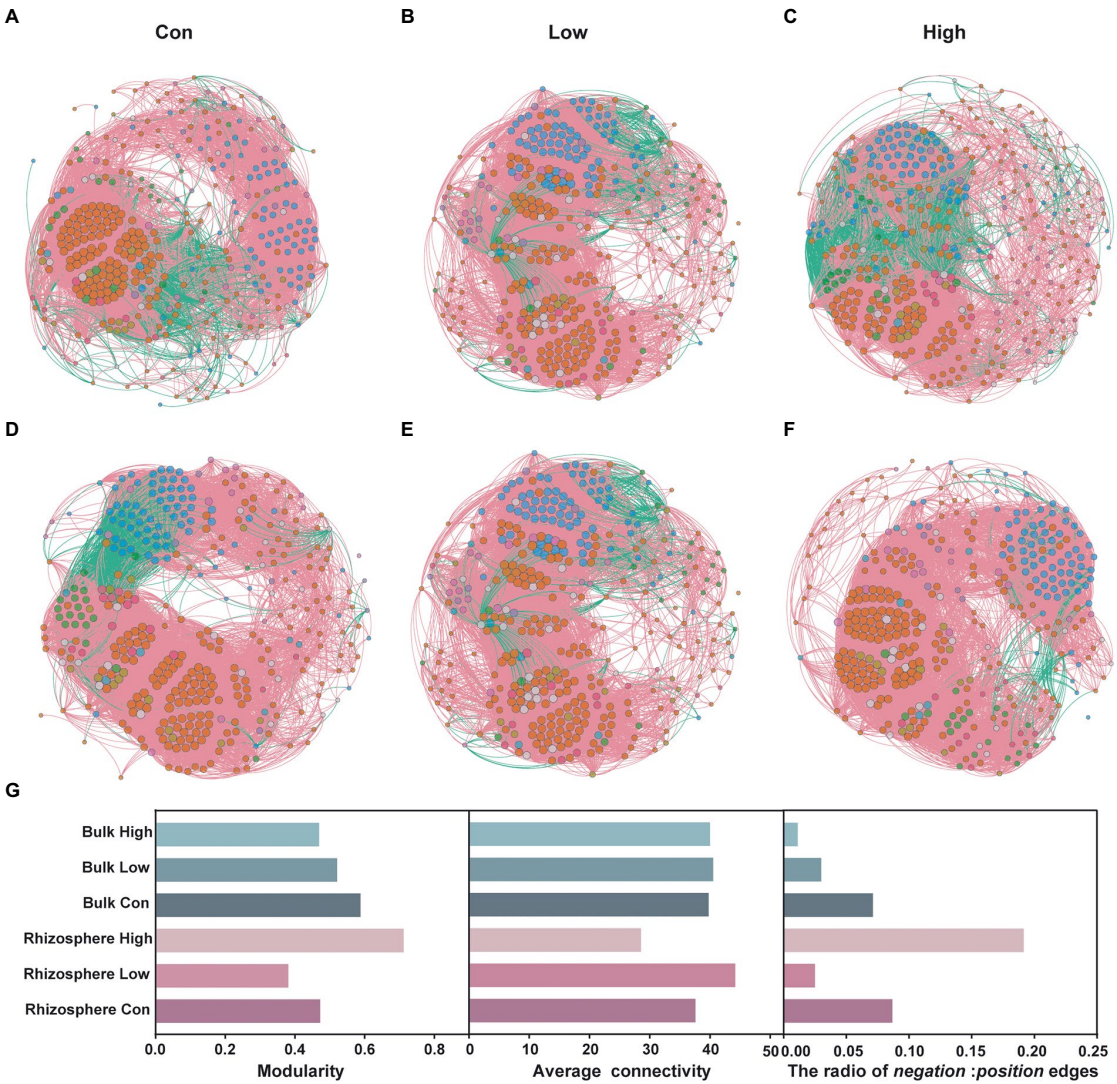
Effects of abamectin on the stability of bulk and rhizosphere microbial communities. **(A–C)** Co-occurrence patterns of genera in the control, low and high of abamectin treatments in rhizosphere. **(D–F)** Co-occurrence patterns of genera in the control, low and high of abamectin treatments in bulk. Nodes are colored according to the main phyla, and node size represents the number of connections. **(G)** Modularity, average connectivity, and the ratio of *negative*:*positive* edges of co-occurrence networks.

Co-occurrence networks, which were based on Spearman’s rank correlation coefficients between genera, showed clear differences between the microbial communities with or without abamectin treatment (Figures 2A–F). There were decreases in modularity and the ratio of *negative*:*positive* edges of the bulk microbial communities after abamectin treatment, compared to the control (Figure 2G). In the rhizosphere microbial communities, modularity, and the ratio of *negative*:*positive* edges first decreased and then increased as the concentration of the abamectin increased, with the opposite of average connectivity (Figure 2G). The trend of the average connectivity of networks in bulk microbial communities was consistent with the trend in the rhizosphere (Figure 2G).

### Effects of abamectin on opportunistic human pathogens

**Figure 3:**
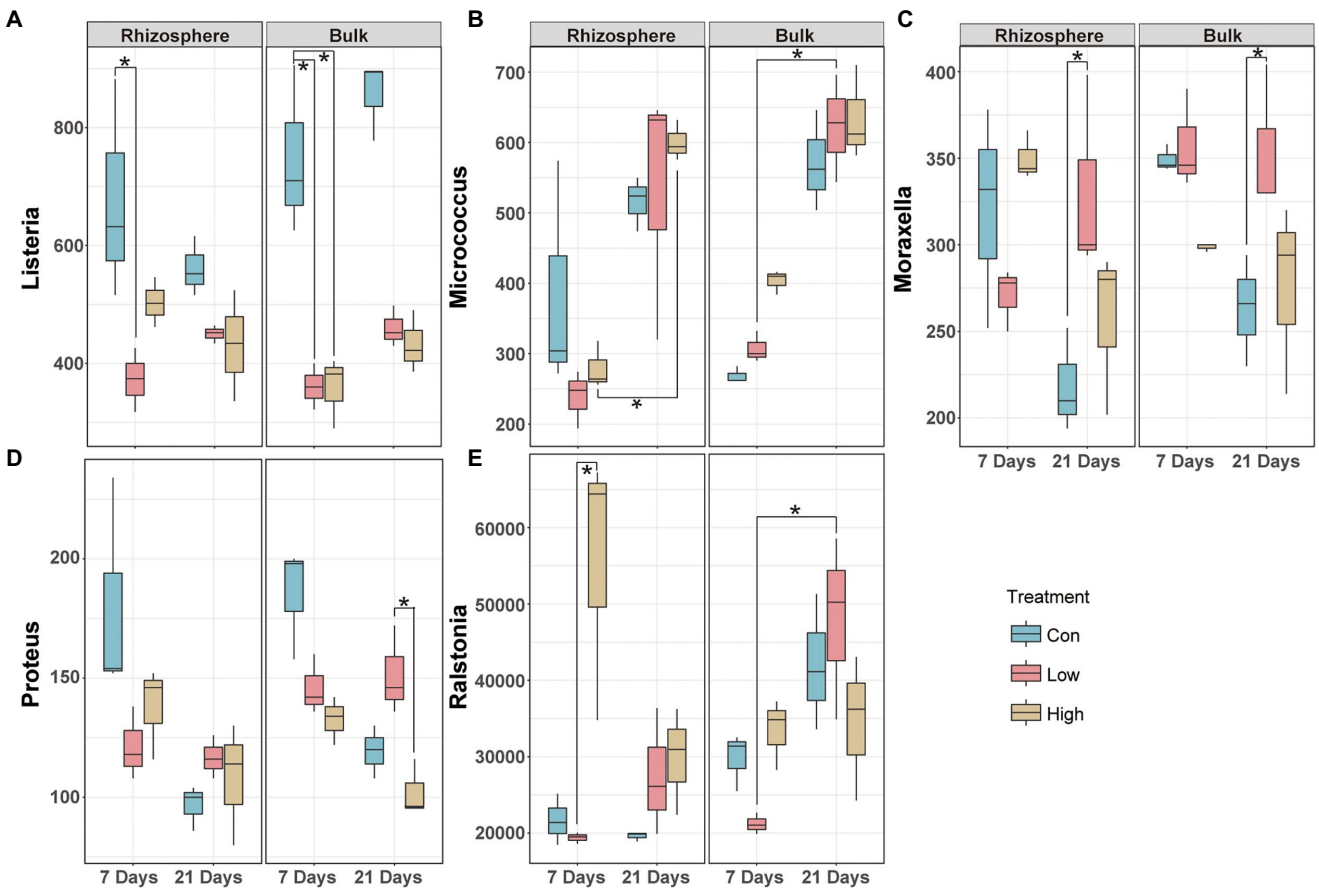
Effects of abamectin on composition of opportunistic human pathogens. Relative abundance of pathogens with or without abamectin treatments at 7 and 21 days. **A-E:** The five pathogens are marked in all samples. The significant difference evaluated by Kruskal–Wallis’s test (*represent *p* < 0.05).

Sustained inputs of abamectin elicited changes in the structure of the soil microbial community, including the prevalence of opportunistic human pathogens that pose potential risks to human health. The 67 genera in our dataset were screened after matching the opportunistic pathogenic database. The pathogens that were altered significantly in the rhizosphere and bulk microbial communities were different (Supplementary Figure S3). *Moraxella*, *Listeria*, *Ralstonia*, *Micrococcus*, *Proteus* varied significantly in both the rhizosphere and the bulk microbial communities (Supplementary Figure S3). In the rhizosphere, the abundance of the pathogens was decreased or did not change significantly except *Ralstonia* after treatment during 7 and 21 days (Figure 3). Different from the observations in the rhizosphere environment, we observed that the impact of abamectin treatment on pathogens in the bulk micro-ecosystem after 21 days of exposure was generally greater than after 7 days (Figure 3). *Ralstonia* is not only a potential human pathogen but also a typical soil-borne pathogen (Yuan et al., 2020). The treatment with a low concentration of abamectin reduced the risk of *Rolstonia* but enriched *Ralstonia* in the high concentration treatment after 7 days of exposure (Figure 3E).

### Effects of abamectin on potential functions of soil microbial communities

**Figure 4:**
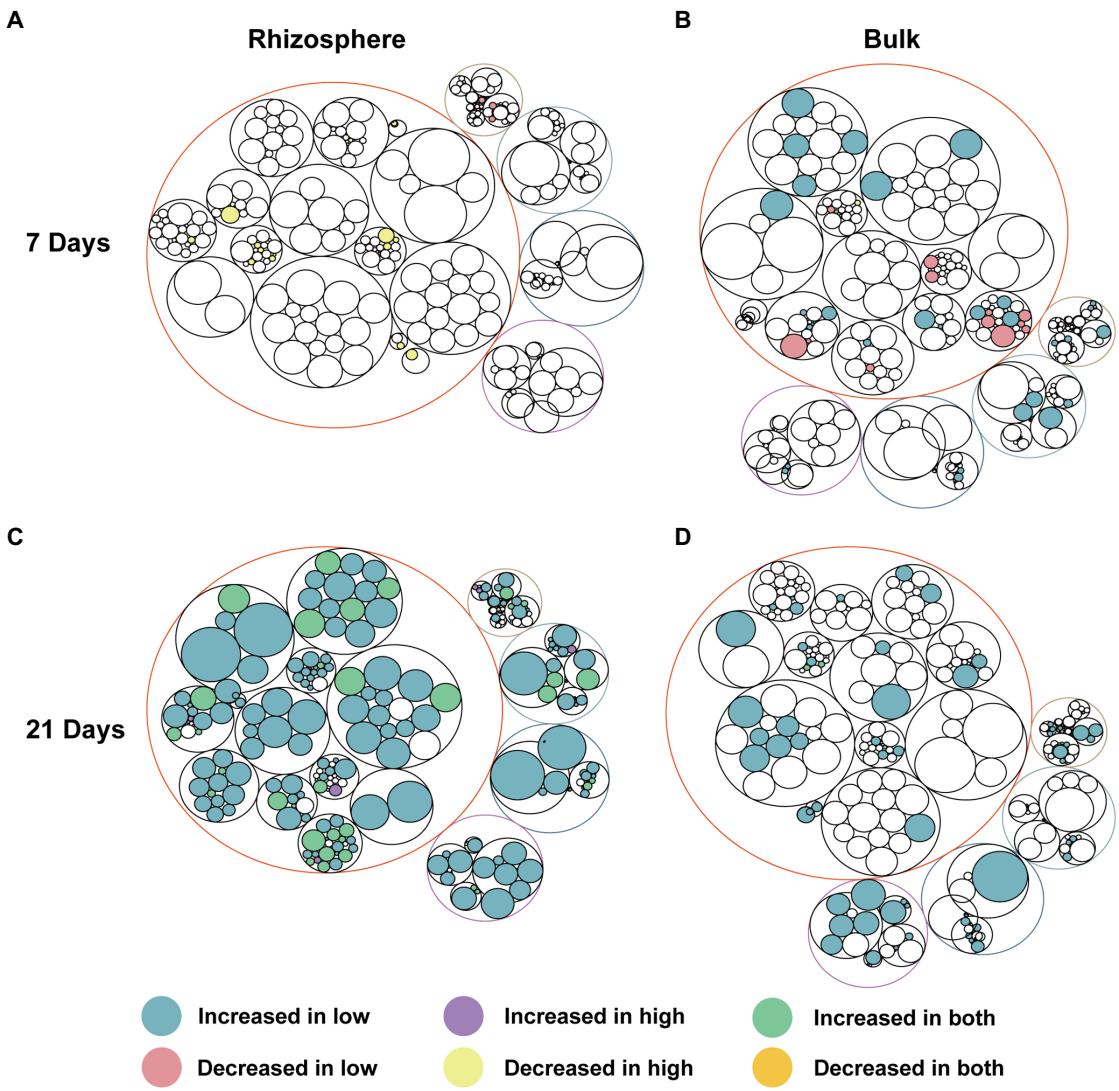
Functional pathways of the microbial communities altered after abamectin treatments. The different colors of the outermost layer represented different categories of metabolic pathway functions. The outermost circles represent the pathways at the KEGG level 1, the deepest circles represent the functional pathways at KEGG level 3. Pathways showing significant difference (*p <* 0.05) between the control and abamectin treatments are the legends showed. Circle size represents the average relative abundance of pathways in all samples. **(A,C)** Represent the functional pathways in rhizosphere at the 7 and 21 days. **(B,D)** Represent the functional pathways in bulk at the 7 and 21 days.

As we expected, the microbial community function potential of bulk and rhizosphere was perturbed by abamectin treatments (Figure 4) due to the shift in microbial composition (Figures 1E–G). Almost every functional pathway was affected after abamectin treatment, albeit to a low degree (Figure 4; Supplementary Figure S4). Based on the KEGG database annotations, the distribution of the abundances of microbial functional genes was similar regardless of whether they originated from bulk or rhizosphere soils. The microbial functions in bulk and rhizosphere were mainly annotated under “Metabolism,” “Genetic Information Processing,” “Environmental Information Processing,” “Cellular Processes,” “Organismal Systems” categories (Figure 4).

After 7 days of treatment, most metabolic functions, such as xenobiotics biodegradation and metabolism, biosynthesis of other secondary metabolites, lipid metabolism, and glycan biosynthesis and metabolism, had decreased by abamectin treatment in the bulk as well as the rhizosphere microbial communities (Figures 4A,B). In contrast, most metabolism-related functions become enriched after 21 days of abamectin treatments, especially the rhizosphere microbial community (Figures 4C,D). Additionally, some functions that were enriched were associated with cellular processes, genetic information processing, environmental information processing, and organismal systems for 21 days (Figures 4B,D).

In our study, after abamectin treatments, the cellular processes, signal transduction, and metabolic processes associated with the functional potential of bulk and rhizosphere microorganisms were decreased for 7 days and recovered for 21 days. These decreases in the essential functions of the microbial community indicated that abamectin treatments negatively affected the bulk and rhizosphere soil ecosystems, but with a different pattern. This different pattern may be attributed to the fact that xenobiotics treatment affected the release of plant root exudates (Lu et al., 2018; Feng et al., 2021; Qu et al., 2021). The effect of abamectin on root exudates interfered with the functional potential of rhizosphere microorganisms, such as signal transduction, the release of signaling molecules (Ke et al., 2020).

During 21 days of exposure, the significant increases in bulk and rhizosphere microbial functional genes related to xenobiotics biodegradation, xenobiotics metabolism, and carbohydrate metabolism (Supplementary Table S1). Degradation of pesticides and nutrient utilization of microbial communities are strongly correlated with carbohydrate metabolism, energy metabolism, biosynthesis of other secondary metabolites, xenobiotics biodegradation, and metabolism (Supplementary Table S1; Zheng B. et al., 2022; Zheng X. et al., 2022). Since abamectin can be rapidly degraded to 8a-hydroxyavermectin B1a and the corresponding ring-opened aldehyde (Halley et al., 1993; Bai and Ogbourne, 2016), these low-toxicity degradation products of abamectin may be taken up as a carbon source for microorganisms. Thus after 21 days, the negative effect of abamectin on the functional potential of the bulk and rhizosphere microbial communities diminished.

### Effects of abamectin on ARGs dissemination

**Figure 5:**
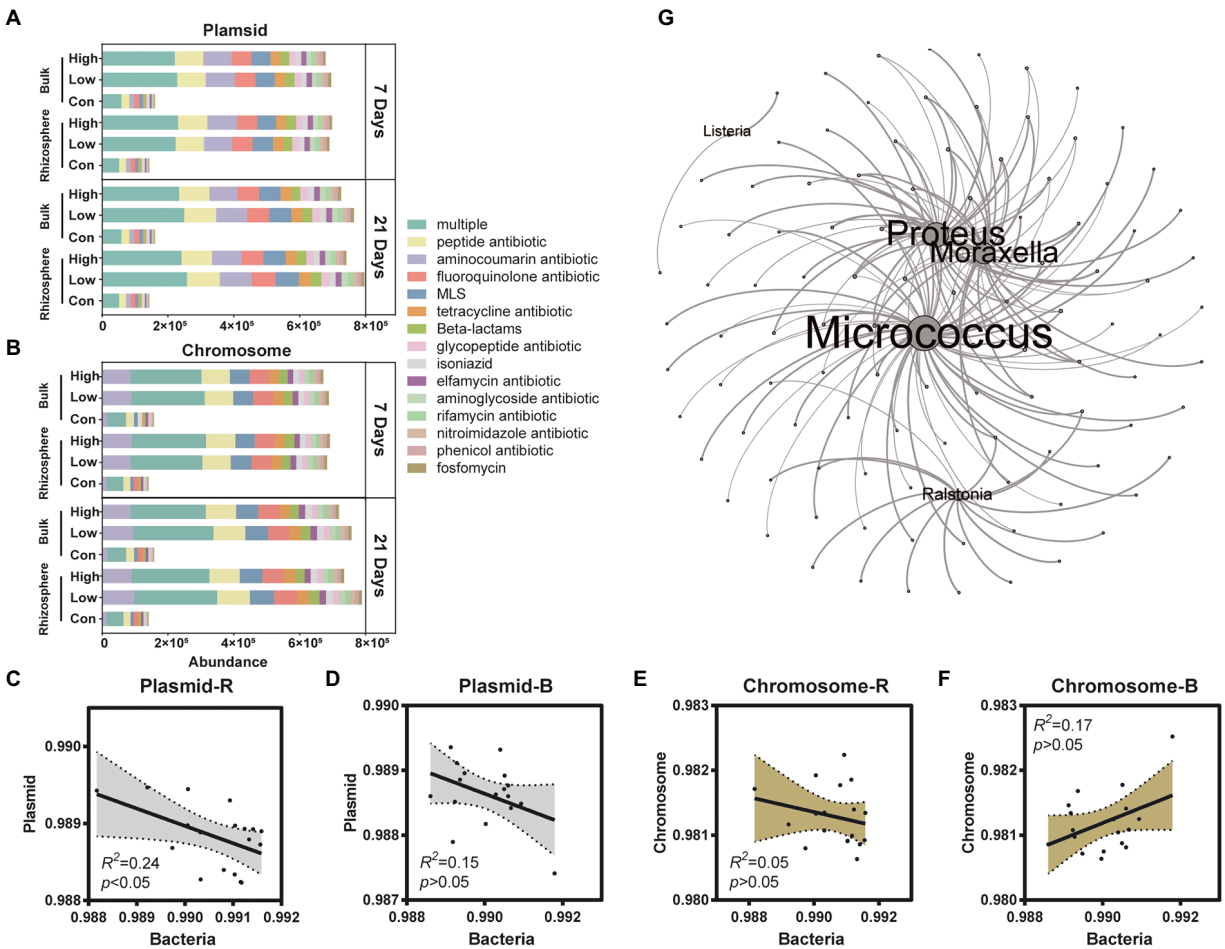
Effects of abamectin treatments on ARGs. **(A,B)** Compositions of ARGs in plasmids and chromosomes, respectively. Only ARG types detected in all samples are shown. **(C–F)** Liner regression analysis of the relative abundance of bacteria and ARGs carried by plasmid or chromosome. Dotted line shows 95% confidence intervals. **(G)** The co-occurrence of pathogens and AGRs, the size of nodes represented the correlation coefficient of pathogens and ARGs.

ARGs were widely distributed in the soil, and the soil microorganisms can produce, resist, or degrade antibiotics and even catabolize them (Crofts et al., 2018; Zhang et al., 2019). The effect of the antibiotic pesticide abamectin on ARGs dissemination associated with bulk and rhizosphere microorganisms is unknown until now. Four hundred and thirty-three ARGs were detected in control and abamectin treatments, and abamectin found to affect the transfer and prevalence of ARGs. Horizontal gene transfer (HGT) and vertical gene transfer (VGT) potentials of ARGs were further determined: 386 ARGs were carried by the plasmid, whereas only 350 ARGs were carried on the chromosome (Supplementary Figure S5). In addition, both the plasmid and the chromosome carried 325 ARGs, 47 of which could not be clearly attributed to either group (Supplementary Figure S5). We also determined the composition of ARGs and found that ARGs carried by chromosomes and plasmids tended to confer multidrug resistance (Figures 5A,B). After 7 days, the total abundance of the main ARGs on chromosomes and plasmids increased following abamectin treatments both in the rhizosphere and bulk soil. However, unlike the variations in diversity and functions of microorganisms, the total abundance of the main ARGs still increased in the rhizosphere after abamectin treatment during the 21 days (Figures 5A,B). The total abundance of ARGs on plasmids was linked to bacterial abundance that had increased, but there was no significant linear relationship between the total abundance of ARGs on chromosomes and the bacterial abundance (Figures 5C–F).

Inputs of abamectin elicited variations in the structure of the soil microbial communities, including the prevalence of opportunistic human pathogens and soil-borne organisms that are harmful to human health or ecological risks (Zhang et al., 2022c). Co-occurrence analysis of ARGs and significant pathogens allowed to detect the most potential antibiotic-resistant pathogen (*Micrococcus*; Figure 5G). In contrast, *Micrococcus* was inhibited in the rhizosphere soil but enriched in the bulk soil after 7 days of exposure (Figure 3B). *Micrococcus* were the major genera associated with ARGs. The abundance of *Micrococcus* decreased due to low metabolic activity under environmental stress and revived after settling in suitable environments (He et al., 2020).

## Discussion

The Shannon index of the rhizosphere microorganisms increased significantly after abamectin treatment can be explained by the fact that plants resist abiotic stress by increasing the diversity of the rhizosphere microbial community (Yu et al., 2022). Soil microbial communities were disrupted after 7 days and gradually recovered the microbial succession after 21 days of exposure. A recent study reported that the growth regulation period of the microbial community is usually about 15 days (Chen et al., 2022). The structure of the soil microbial community can be altered by some variations, such as metabolic pathways, nutrition-related gene expression, and quorum sensing when facing the different stresses of pesticides (Burns et al., 2013). The significant differences in dominant phyla of rhizosphere and bulk microorganisms showed that rice as the plant host induced selectivity among the phyla. In soil ecosystems, microorganisms as nodes and their relationships as links can be represented as networks, which is fundamental for microbial communities to respond to environmental stress (Montoya et al., 2006; Zhang et al., 2021, 2022b). The network stability was strongly correlated with the network complexity (Yuan, M. M. et al., 2021). Adding average connectivity and modularity increased the complexity of the network (de Vries et al., 2018). The ratio of negative:positive edges negatively correlated with environmental stress (Hernandez et al., 2021). This explains why bulk microbial communities were unstable under the abamectin treatment. However, increased abamectin concentration stimulated plant-microorganism interactions, leading to more network complexity (Yuan, M. M. et al., 2021). An additional theory stated that higher complexity destabilizes rhizosphere ecological systems (MacArthur, 1955).

Consequently, abamectin treatment can affect the microbial community structure and composition, and the microbial community function potential of bulk and rhizosphere is perturbed. Although abamectin is rapidly degraded in soil and has low toxicity to crops (Bai and Ogbourne, 2016), it disrupted the homeostasis of the soil microbial communities after 7 days of exposure. Compared with bulk microorganisms, there were more interactions of plant microorganisms in the root. The application of abamectin may enrich abamectin degradation bacteria, and study in future will identify them for soil remediation. The abundance of *Rolstonia* varied after abamectin treatment suggesting an increase of the risk in plant diseases, such as bacterial wilt, after short-term treatment at high concentrations of abamectin. *Rolstonia* invaded soil microbial networks and disrupted microbial interactions under environmental stress (Li et al., 2021). This is consistent with our results as reported above (Figure 2). Due to the readily degradability of abamectin, soil microbial interactions without the effects of abamectin at 21 days can improve the ability of soil to recover from pathogen infestation (Deng et al., 2021).

The assessment of the risks associated with the impacts of abamectin on the abundance and transfer of ARGs, and pathogens harmful to human and ecological health in soil is urgently required. After abamectin treatments, the diversity, and functional gene abundance of the soil microbial community decreased at 7 days of exposure but recovering after 21 days. Unlike the variations in diversity and functions of microorganisms, the abundance of ARGs was increased not only after 7 days of exposure, but also 21 days after abamectin treatments. In our study, the ARGs was classified as plasmid-carried ARG and chromosome-carried ARG. The HGT cannot be achieved by the chromosome-carried ARGs between bacterial cells (Che et al., 2019). Plasmid-carried ARGs are more threatening to human and soil ecosystem health than chromosome-carried ARGs in bacteria, due to transfers from antibiotic-resistant bacteria (ARB) to non-ARB on plasmids (Jin et al., 2020; Qiu et al., 2022). In our study, the increases in the abundance of ARGs on plasmids were not strongly correlated with bacterial abundance (*R*^2^ = 0.24 and 0.15; Figures 5A–D), which proved that those plasmids were more stable and transferable carriers than bacterial chromosomes. Our results revealed that ARGs were transferred more widely and had more durable effects on soil ecosystem health after abamectin treatments.

## Conclusion

In conclusion, our study concentrated on the effects of abamectin treatments on microbial communities and ARGs dissemination in bulk and rhizosphere soils. Our results showed that even a low concentration of abamectin could disrupt and destabilize the structure of microbial communities, and temporarily decrease the metabolic capability of the soil microbial communities. At the same time, abamectin also promoted the growth of some potential pathogens and accelerated the transfer of ARGs in soil. After 21 days of exposure, the soil microbial communities could recover after abamectin treatments except for ARGs dissemination. These findings highlight the risks of human and soil ecosystem health with the widespread use of abamectin.

## Data availability statement

The data presented in the study are deposited in the NCBI Sequence Read Archive (SRA) database, accession number PRJNA886835, and accession number can be found at: https://www.ncbi.nlm.nih.gov/bioproject/PRJNA886835.

## Author contributions

DQ: methodology, investigation, writing—original draft, and writing—review and editing. NX and QZ: methodology and investigation. WZ: data analysis. YW: data collection. ZZ: investigation and methodology. YY: data collection and validation. TL: software. LS: formal analysis. N-YZ: formal analysis and writing—original draft. WP: writing—review and editing HQ: conceptualization, writing—review and editing, and funding acquisition. All authors contributed to the article and approved the submitted version.

## Funding

This work was financially supported by the National Natural Science Foundation of China (21976161, 21777145, and 41907210) and Open Funding Project of State Key Laboratory of Microbial Metabolism (MMLKF20-05).

## Conflict of interest

The authors declare that the research was conducted in the absence of any commercial or financial relationships that could be construed as a potential conflict of interest.

## Publisher’s note

All claims expressed in this article are solely those of the authors and do not necessarily represent those of their affiliated organizations, or those of the publisher, the editors and the reviewers. Any product that may be evaluated in this article, or claim that may be made by its manufacturer, is not guaranteed or endorsed by the publisher.

## Footnotes

^1^ https://github.com/hyattpd/Prodigal

^2^ https://github.com/bbuchfink/diamond/

^3^ https://www.bode-science-center.com/center/relevant-pathogens-from-a-z.html

## References

[ref1] Bahram M. Hildebrand F. Forslund S. K. Anderson J. L. Soudzilovskaia N. A. Bodegom P. M.. (2018). Structure and function of the global topsoil microbiome. Nature 560, 233–237. doi: 10.1038/s41586-018-0386-6 30069051

[ref2] Bai S. H. Ogbourne S. (2016). Eco-toxicological effects of the avermectin family with a focus on abamectin and ivermectin. Chemosphere 154, 204–214. doi: 10.1016/j.chemosphere.2016.03.113 27058912

[ref3] Bolger A. M. Lohse M. Usadel B. (2014). Trimmomatic: a flexible trimmer for Illumina sequence data. Bioinformatics 30, 2114–2120. doi: 10.1093/bioinformatics/btu170 24695404 PMC4103590

[ref4] Bulgarelli D. Rott M. Schlaeppi K. van Themaat E. V. L. Ahmadinejad N. Assenza F.. (2012). Revealing structure and assembly cues for arabidopsis root-inhabiting bacterial microbiota. Nature 488, 91–95. doi: 10.1038/nature11336 22859207

[ref5] Burns R. G. DeForest J. L. Marxsen J. Sinsabaugh R. L. Stromberger M. E. Wallenstein M. D.. (2013). Soil enzymes in a changing environment: current knowledge and future directions. Soil Biol. Biochem. 58, 216–234. doi: 10.1016/j.soilbio.2012.11.009

[ref6] Che Y. Xia Y. Liu L. Li A.-D. Yang Y. Zhang T. (2019). Mobile antibiotic resistome in wastewater treatment plants revealed by Nanopore metagenomic sequencing. Microbiome 7:44. doi: 10.1186/s40168-019-0663-0 30898140 PMC6429696

[ref7] Chen C. Wang M. Zhu J. Tang Y. Zhang H. Zhao Q.. (2022). Long-term effect of epigenetic modification in plant–microbe interactions: modification of DNA methylation induced by plant growth-promoting bacteria mediates promotion process. Microbiome 10:36. doi: 10.1186/s40168-022-01236-9 35209943 PMC8876431

[ref8] Crofts T. S. Wang B. Spivak A. Gianoulis T. A. Forsberg K. J. Gibson M. K.. (2018). Shared strategies for β-lactam catabolism in the soil microbiome. Nat. Chem. Biol. 14, 556–564. doi: 10.1038/s41589-018-0052-1 29713061 PMC5964007

[ref9] De Vries F. T. Griffiths R. I. Bailey M. Craig H. Girlanda M. Gweon H. S.. (2018). Soil bacterial networks are less stable under drought than fungal networks. Nat. Commun. 9:3033. doi: 10.1038/s41467-018-05516-7 30072764 PMC6072794

[ref10] Deng X. Zhang N. Shen Z. Zhu C. Liu H. Xu Z.. (2021). Soil microbiome manipulation triggers direct and possible indirect suppression against Ralstonia solanacearum and fusarium oxysporum. NPJ Biofilms Microbiomes 7, 1–10. doi: 10.1038/s41522-021-00204-9 33846334 PMC8041757

[ref11] Dennis P. G. Miller A. J. Hirsch P. R. (2010). Are root exudates more important than other sources of rhizodeposits in structuring rhizosphere bacterial communities? FEMS Microbiol. Ecol. 72, 313–327. doi: 10.1111/j.1574-6941.2010.00860.x 20370828

[ref12] Edwards J. Johnson C. Santos-Medellin C. Lurie E. Podishetty N. K. Bhatnagar S.. (2015). Structure, variation, and assembly of the root-associated microbiomes of rice. Proc. Natl. Acad. Sci. U. S. A. 112, E911–E920. doi: 10.1073/pnas.1414592112 25605935 PMC4345613

[ref13] El-Saber Batiha G. Alqahtani A. Ilesanmi O. B. Saati A. A. El-Mleeh A. Hetta H. F.. (2020). Avermectin derivatives, pharmacokinetics, therapeutic and toxic dosages, mechanism of action, and their biological effects. Pharmaceuticals 13:8. doi: 10.3390/ph13080196 32824399 PMC7464486

[ref14] Feng L. Xu N. Qu Q. Zhang Z. Ke M. Lu T.. (2021). Synergetic toxicity of silver nanoparticle and glyphosate on wheat (*Triticum aestivum L.*). Sci. Total Environ. 797:149200. doi: 10.1016/j.scitotenv.2021.149200 34303973

[ref15] Fu L. Niu B. Zhu Z. Wu S. Li W. (2012). CD-HIT: accelerated for clustering the next-generation sequencing data. Bioinformatics 28, 3150–3152. doi: 10.1093/bioinformatics/bts565 23060610 PMC3516142

[ref16] Ge T. Yuan H. Zhu H. Wu X. Nie S. Liu C.. (2012). Biological carbon assimilation and dynamics in a flooded rice – soil system. Soil Biol. Biochem. 48, 39–46. doi: 10.1016/j.soilbio.2012.01.009

[ref17] Halley B. A. VandenHeuvel W. J. A. Wislocki P. G. (1993). Environmental effects of the usage of avermectins in livestock. Vet. Parasitol. 48, 109–125. doi: 10.1016/0304-4017(93)90149-H 8346626

[ref18] He Y. Gao C. Cao M. Chen W. Huang L. Zhou W.. (2007). Survey of susceptibilities to monosultap, triazophos, fipronil, and abamectin in chilo suppressalis (lepidoptera: Crambidae). J. Econ. Entomol. 100, 1854–1861. doi: 10.1093/jee/100.6.1854 18232403

[ref19] He P. Wu Y. Huang W. Wu X. Lv J. Liu P.. (2020). Characteristics of and variation in airborne ARGs among urban hospitals and adjacent urban and suburban communities: a metagenomic approach. Environ. Int. 139:105625. doi: 10.1016/j.envint.2020.105625 32251897

[ref20] Hernandez D. J. David A. S. Menges E. S. Searcy C. A. Afkhami M. E. (2021). Environmental stress destabilizes microbial networks. ISME J. 15, 1722–1734. doi: 10.1038/s41396-020-00882-x 33452480 PMC8163744

[ref21] Huang B. Li J. Wang Q. Guo M. Yan D. Fang W.. (2018). Effect of soil fumigants on degradation of abamectin and their combination synergistic effect to root-knot nematode. PLoS One 13:e0188245. doi: 10.1371/journal.pone.0188245 29889848 PMC5995350

[ref22] Huson D. H. Auch A. F. Qi J. Schuster S. C. (2007). MEGAN analysis of metagenomic data. Genome Res. 17, 377–386. doi: 10.1101/gr.5969107 17255551 PMC1800929

[ref23] Jiao C. Chen L. Sun C. Jiang Y. Zhai L. Liu H.. (2020). Evaluating national ecological risk of agricultural pesticides from 2004 to 2017 in China. Environ. Pollut. 259:113778. doi: 10.1016/j.envpol.2019.113778 31918127

[ref24] Jin M. Liu L. Wang D. Yang D. Liu W. Yin J.. (2020). Chlorine disinfection promotes the exchange of antibiotic resistance genes across bacterial genera by natural transformation. ISME J. 14, 1847–1856. doi: 10.1038/s41396-020-0656-9 32327733 PMC7305130

[ref25] Ke M. Li Y. Qu Q. Ye Y. Peijnenburg W. J. G. M. Zhang Z.. (2020). Offspring toxicity of silver nanoparticles to Arabidopsis thaliana flowering and floral development. J. Hazard. Mater. 386:121975. doi: 10.1016/j.jhazmat.2019.121975 31884364

[ref26] Kepler R. M. Epp Schmidt D. J. Yarwood S. A. Cavigelli M. A. Reddy K. N. Duke S. O.. (2020). Soil microbial communities in diverse agroecosystems exposed to the herbicide glyphosate. Appl. Environ. Microbiol. 86:e01744-19. doi: 10.1128/AEM.01744-19 31836576 PMC7028976

[ref27] Kolar L. Kožuh Eržen N. Hogerwerf L. van Gestel C. A. M. (2008). Toxicity of abamectin and doramectin to soil invertebrates. Environ. Pollut. 151, 182–189. doi: 10.1016/j.envpol.2007.02.011 17434247

[ref28] Li P. Liu M. Li G. Liu K. Liu T. Wu M.. (2021). Phosphorus availability increases pathobiome abundance and invasion of rhizosphere microbial networks by Ralstonia. Environ. Microbiol. 23, 5992–6003. doi: 10.1111/1462-2920.15696 34347357

[ref29] Li D. Liu C.-M. Luo R. Sadakane K. Lam T.-W. (2015). MEGAHIT: an ultra-fast single-node solution for large and complex metagenomics assembly via succinct de Bruijn graph. Bioinformatics 31, 1674–1676. doi: 10.1093/bioinformatics/btv033 25609793

[ref30] Lu T. Ke M. Lavoie M. Jin Y. Fan X. Zhang Z.. (2018). Rhizosphere microorganisms can influence the timing of plant flowering. Microbiome 6:231. doi: 10.1186/s40168-018-0615-0 30587246 PMC6307273

[ref31] Lundberg D. S. Lebeis S. L. Paredes S. H. Yourstone S. Gehring J. Malfatti S.. (2012). Defining the core Arabidopsis thaliana root microbiome. Nature 488, 86–90. doi: 10.1038/nature11237 22859206 PMC4074413

[ref32] MacArthur R. (1955). Fluctuations of animal populations and a measure of community stability. Ecology 36, 533–536. doi: 10.2307/1929601

[ref33] Majer H. M. Ehrlich R. L. Ahmed A. Earl J. P. Ehrlich G. D. Beld J. (2021). Whole genome sequencing of Streptomyces actuosus ISP-5337, Streptomyces sioyaensis B-5408, and Actinospica acidiphila B-2296 reveals secondary metabolomes with antibiotic potential. Biotechnol. Rep. 29:e00596. doi: 10.1016/j.btre.2021.e00596 PMC7893419 33643857

[ref34] Marrone P. G. (2014). “The market and potential for biopesticides,” in Biopesticides: State of the art and future opportunities, ACS symposium series (NW, Washington, DC: American Chemical Society), 245–258.

[ref35] Marrone P. G. (2019). Pesticidal natural products – status and future potential. Pest Manag. Sci. 75, 2325–2340. doi: 10.1002/ps.5433 30941861

[ref36] Montoya J. M. Pimm S. L. Solé R. V. (2006). Ecological networks and their fragility. Nature 442, 259–264. doi: 10.1038/nature04927 16855581

[ref37] Qiu D. Ke M. Zhang Q. Zhang F. Lu T. Sun L.. (2022). Response of microbial antibiotic resistance to pesticides: an emerging health threat. Sci. Total Environ. 850:158057. doi: 10.1016/j.scitotenv.2022.158057 35977623

[ref38] Qu Q. Li Y. Zhang Z. Cui H. Zhao Q. Liu W.. (2021). Effects of S-metolachlor on wheat (Triticum aestivum L.) seedling root exudates and the rhizosphere microbiome. J. Hazard. Mater. 411:125137. doi: 10.1016/j.jhazmat.2021.125137 33858101

[ref39] Qu Q. Zhang Z. Peijnenburg W. J. G. M. Liu W. Lu T. Hu B.. (2020). Rhizosphere microbiome assembly and its impact on plant growth. J. Agric. Food Chem. 68, 5024–5038. doi: 10.1021/acs.jafc.0c00073 32255613

[ref40] Ramirez K. S. Knight C. G. de Hollander M. Brearley F. Q. Constantinides B. Cotton A.. (2018). Detecting macroecological patterns in bacterial communities across independent studies of global soils. Nat. Microbiol. 3, 189–196. doi: 10.1038/s41564-017-0062-x 29158606

[ref41] Ray P. Lakshmanan V. Labbé J. L. Craven K. D. (2020). Microbe to microbiome: a paradigm shift in the application of microorganisms for sustainable agriculture. Front. Microbiol. 11:622926. doi: 10.3389/fmicb.2020.622926 33408712 PMC7779556

[ref42] Tramberend S. Fischer G. Bruckner M. van Velthuizen H. (2019). Our common cropland: quantifying global agricultural land use from a consumption perspective. Ecol. Econ. 157, 332–341. doi: 10.1016/j.ecolecon.2018.12.005

[ref43] Wall D. H. Nielsen U. N. Six J. (2015). Soil biodiversity and human health. Nature 528, 69–76. doi: 10.1038/nature15744 26595276

[ref44] Wittwer R. A. Franz B. S. Kyle H. Sofia H. Lima Ruy A. A. Viviana L.. (2021). Organic and conservation agriculture promote ecosystem multifunctionality. Science. Advances 7:eabg6995. doi: 10.1126/sciadv.abg6995 PMC8378818 34417179

[ref45] Xu N. Qu Q. Zhang Z. Yuan W. Cui H. Shen Y.. (2020). Effects of residual S-metolachlor in soil on the phyllosphere microbial communities of wheat (*Triticum aestivum* L.). Sci. Total Environ. 748:141342. doi: 10.1016/j.scitotenv.2020.141342 32818888

[ref46] Yu Y. Zhang Q. Zhang Z. Xu N. Li Y. Jin M.. (2022). Assessment of residual chlorine in soil microbial community using metagenomics. Soil Ecol. Lett. 5, 66–78. doi: 10.1007/s42832-022-0130-x PMC8889059 40477893

[ref47] Yuan M. M. Guo X. Wu L. Zhang Y. Xiao N. Ning D.. (2021). Climate warming enhances microbial network complexity and stability. Nat. Clim. Chang. 11, 343–348. doi: 10.1038/s41558-021-00989-9

[ref48] Yuan S. Linquist B. A. Wilson L. T. Cassman K. G. Stuart A. M. Pede V.. (2021). Sustainable intensification for a larger global rice bowl. Nat. Commun. 12:7163. doi: 10.1038/s41467-021-27424-z 34887412 PMC8660894

[ref49] Yuan J. Wen T. Zhang H. Zhao M. Penton C. R. Thomashow L. S.. (2020). Predicting disease occurrence with high accuracy based on soil macroecological patterns of fusarium wilt. ISME J. 14, 2936–2950. doi: 10.1038/s41396-020-0720-5 32681158 PMC7784920

[ref50] Zhang Q. Yu Y. Jin M. Deng Y. Zheng B. Lu T.. (2022). Oral azoxystrobin driving the dynamic change in resistome by disturbing the stability of the gut microbiota of Enchytraeus crypticus. J. Hazard. Mater. 423:127252. doi: 10.1016/j.jhazmat.2021.127252 34844364

[ref51] Zhang Z. Zhang Q. Cui H. Li Y. Xu N. Lu T.. (2022a). Composition identification and functional verification of bacterial community in disease-suppressive soils by machine learning. Environ. Microbiol. 24, 3405–3419. doi: 10.1111/1462-2920.15902 35049096

[ref52] Zhang Q. Zhang Z. Lu T. Yu Y. Penuelas J. Zhu Y.-G.. (2021). Gammaproteobacteria, a core taxon in the guts of soil fauna, are potential responders to environmental concentrations of soil pollutants. Microbiome 9:196. doi: 10.1186/s40168-021-01150-6 34593032 PMC8485531

[ref53] Zhang Z. Zhang Q. Lu T. Zhang J. Sun L. Hu B.. (2022b). Residual chlorine disrupts the microbial communities and spreads antibiotic resistance in freshwater. J. Hazard. Mater. 423:127152. doi: 10.1016/j.jhazmat.2021.127152 34537643 PMC9758890

[ref54] Zhang Z. Zhang Q. Wang T. Xu N. Lu T. Hong W.. (2022c). Assessment of global health risk of antibiotic resistance genes. Nat. Commun. 13:1553. doi: 10.1038/s41467-022-29283-8 35322038 PMC8943045

[ref55] Zhang Q. Zhu D. Ding J. Zheng F. Zhou S. Lu T.. (2019). The fungicide azoxystrobin perturbs the gut microbiota community and enriches antibiotic resistance genes in Enchytraeus crypticus. Environ. Int. 131:104965. doi: 10.1016/j.envint.2019.104965 31284112

[ref56] Zheng X. Jahn M. T. Sun M. Friman V.-P. Balcazar J. L. Wang J.. (2022). Organochlorine contamination enriches virus-encoded metabolism and pesticide degradation associated auxiliary genes in soil microbiomes. ISME J. 16, 1397–1408. doi: 10.1038/s41396-022-01188-w 35039616 PMC9038774

[ref57] Zheng B. Zhao Q. Feng L. Zhang Z. Zhang Q. Zhang F.. (2022). Regulative effect of imazethapyr on arabidopsis thaliana growth and rhizosphere microbial community through multiple generations of culture. Plant and Soil 473, 625–637. doi: 10.1007/s11104-022-05318-3

