# Supplementary material for: Negative effects of abamectin on soil microbial communities in the short term
Source: Front Microbiol. 2022 Dec 5;13:1053153. doi: 10.3389/fmicb.2022.1053153 (PMC9760678; doi:10.3389/fmicb.2022.1053153)
Supplement: Supplementary file 1 [file Data_Sheet_1.docx]

# Abamectin disrupts the microbial communities and spreads antibiotic resistance genes in soil

Nuohan Xu^1^, Qi Zhang^1^, Wenya Zhou^2^, Yan Wang^1^, Zhenyan Zhang^1^, Yitian Yu^1^, Tao Lu^1^, Liwei Sun^1^, Ningyi Zhou^3^, W.J.G.M. Peijnenburg^4, 5^, Haifeng Qian^1,^ ^[[1]](#footnote-1)^*

1. College of Environment, Zhejiang University of Technology, 310032, Hangzhou, Zhejiang, P. R. of China

2. College of Environment and Ecology, Xiamen University, 361000, Xiamen, Fujian, P. R. of China

3. State Key Laboratory of Microbial Metabolism, and School of Life Science & Biotechnology, Shanghai Jiao Tong University, 200240, Shanghai, P. R. of China

4. Institute of Environmental Sciences (CML), Leiden University, RA Leiden 2300, the Netherlands

5. National Institute of Public Health and the Environment (RIVM), Center for Safety of Substances and Products, BA Bilthoven 3720, the Netherlands


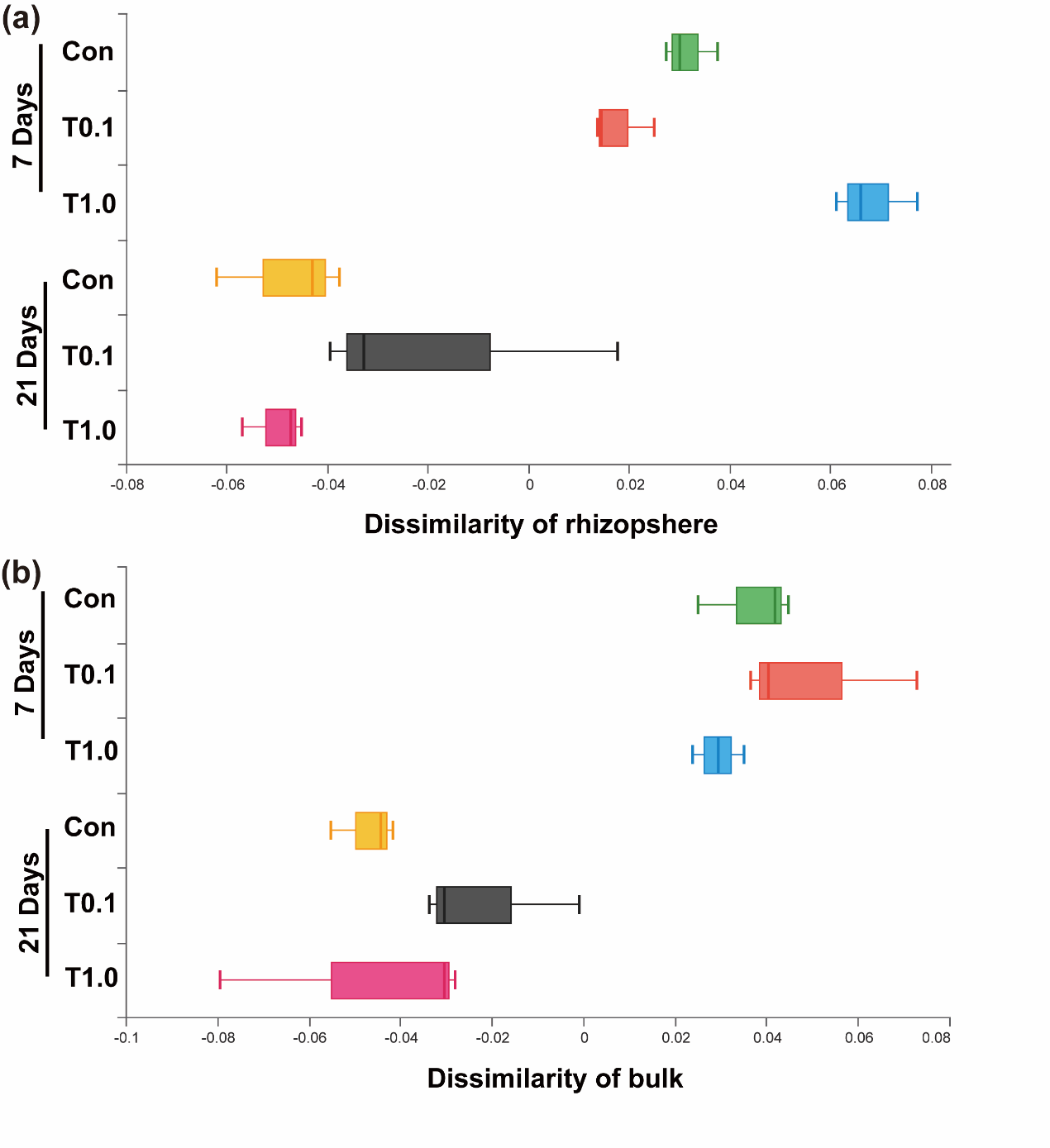


**Figure S1.** The dissimilarity of soil microbial contaminants in bulk and rhizosphere.


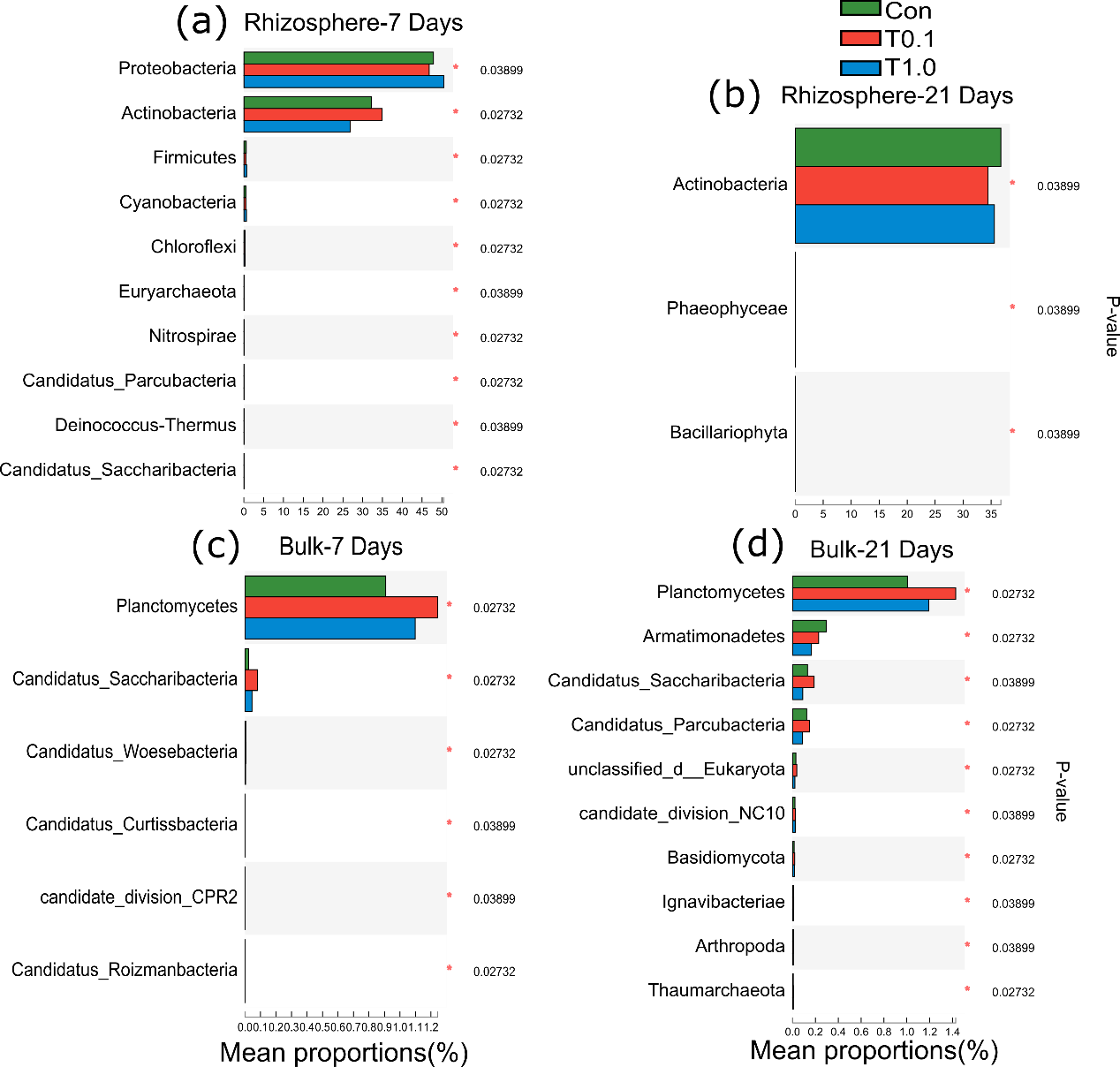


**Figure S2.** Overview of significant differences across phyla among the top 10.

* Represent statistically significant differences at *p* < 0.05 from Kruskal-Wallis’s test.


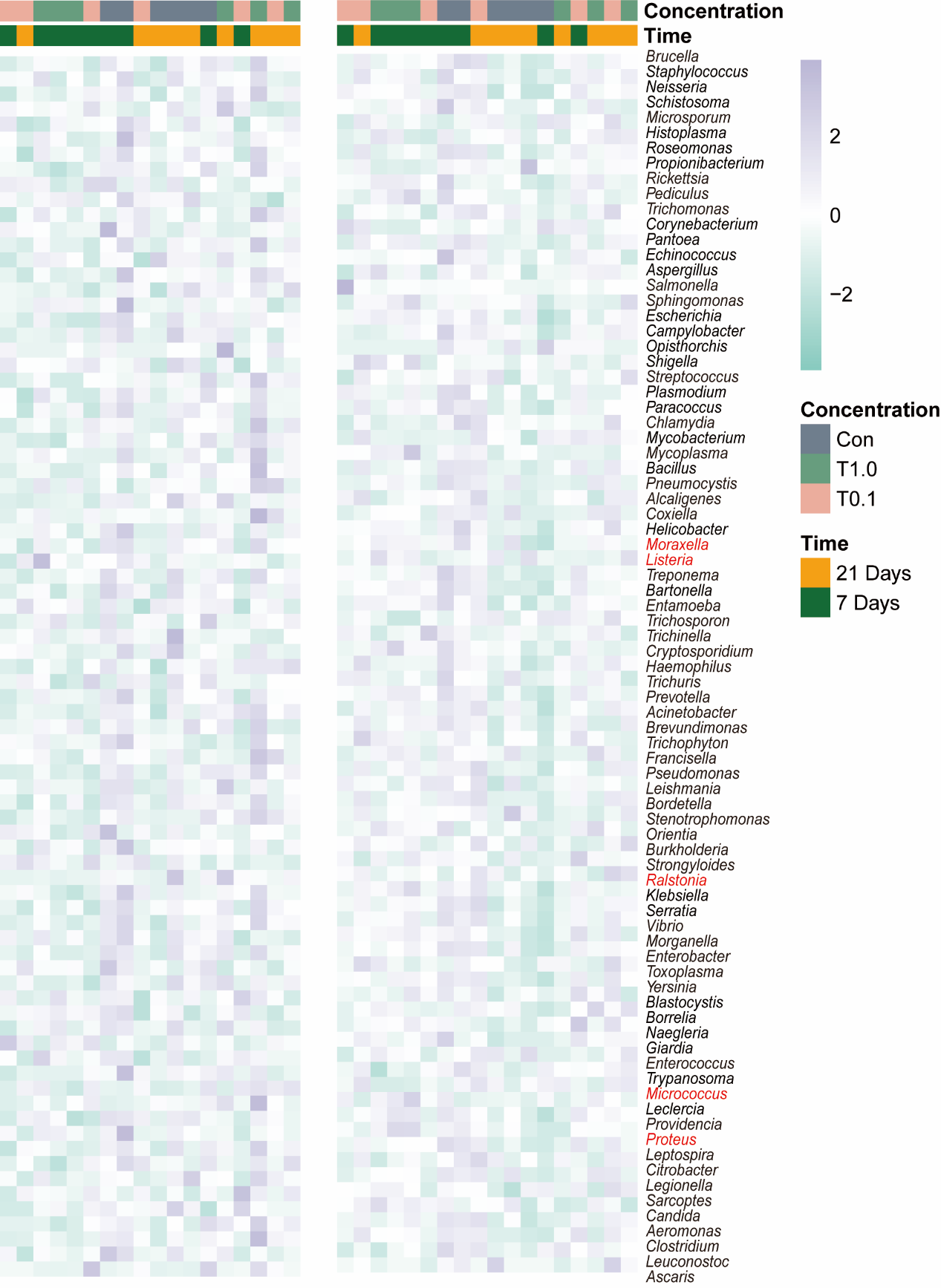


**Figure S3.** The composition of opportunistic human pathogens in all samples. The pathogens marked with red ink represent the significant differences at *p* < 0.05 from Kruskal-Wallis’s test in all samples.


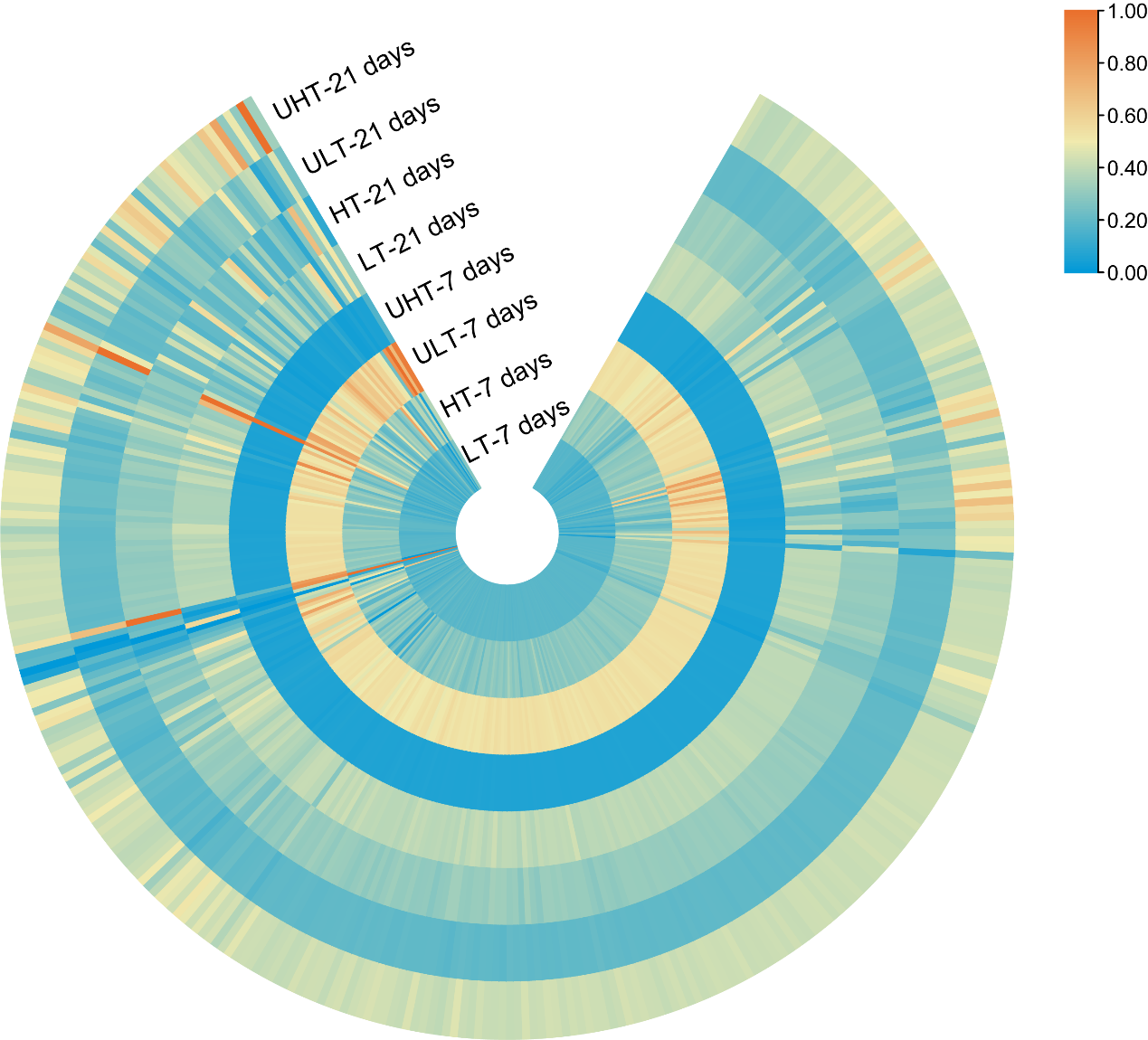


**Figure S4.** The functional pathways of all samples in both bulk and rhizosphere at KEGG level 3. “UHT”, “ULT”, “HT”, “LT” represent the radio of *high abamectin treatment*:*control treatment* in bulk soil, the radio of *low abamectin treatment*:*control treatment* in bulk soil, the radio of *high abamectin treatment*:*control treatment* in rhizosphere soil, and the radio of *low abamectin treatment*:*control treatment* in rhizosphere soil, respectively.


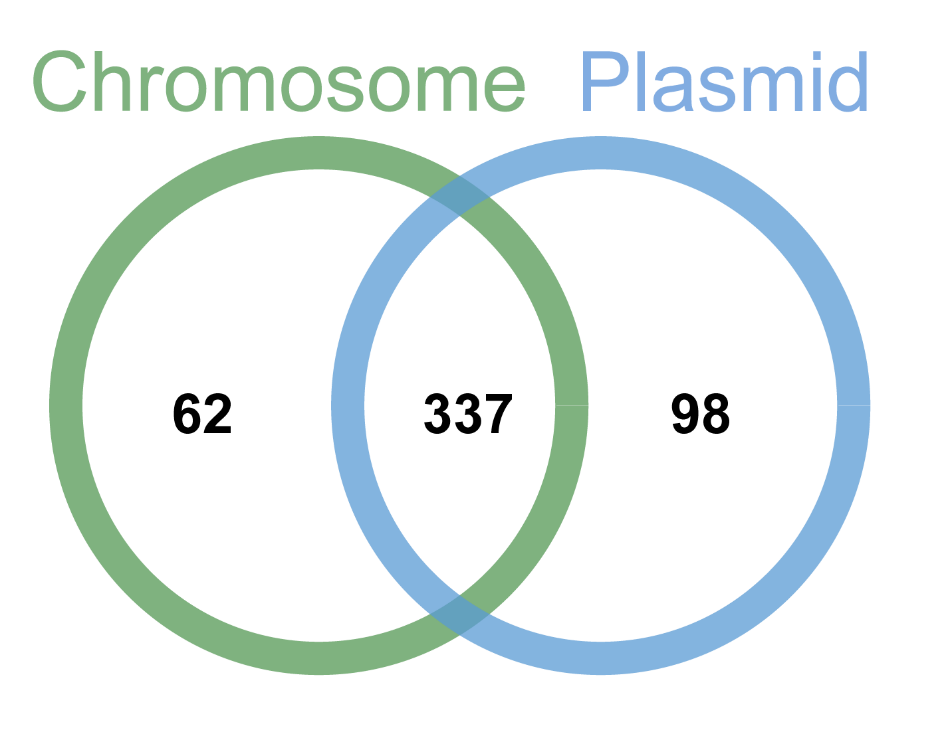


**Figure S5.** Venn diagram of shared and unique ARGs observed in plasmid and chromosome.

1. * Correspondence Author: E-mail: [hfqian@zjut.edu.cn](mailto:hfqian@zjut.edu.cn) (Qian H.). [↑](#footnote-ref-1)
